# Imagem Cardiovascular em Cardiopatias Congênitas: Por que não Alavancar Novas Modalidades de Imagem?

**DOI:** 10.36660/abc.20201347

**Published:** 2021-02-19

**Authors:** Vitor Guerra

**Affiliations:** 1 Hospital for Sick ChildrenDepartment of PediatricsUniversity of TorontoTorontoOntarioCanadáSickkids Hospital/ The Hospital for Sick Children, Department of Pediatrics, University of Toronto, Toronto, Ontario – Canadá

**Keywords:** Cardiopatias Congênitas/cirurgia, Técnicas e Procedimentos de Diagnóstico, Imagem por Ressonância Magnética/métodos, Cateterismo Cardíaco

“Não existe revelação mais nítida da alma de uma sociedade do que a forma como ela trata as suas crianças.”

Nelson Mandela (8 maio 1995)

Mundialmente, estamos vivendo uma era sobrecarregada por doenças cardíacas congênitas (DCC).^1^ Ao tirar uma fotografia instantânea no ano de 2018, vemos que houve 2.944.932 nascidos vivos no Brasil (Tabnet, DATASUS). Considerando a incidência das DCC, cerca de 9 a cada 1.000 nascidos vivos (índice que tem permanecido estável em todos os países e populações), seria de se esperar cerca de 26.000 novos pacientes com alguma forma de DCC em nosso país. Por outro lado, a taxa de sobrevivência mundial para as DCC durante as últimas 3 décadas tem sido próxima a 98%.^2^ Dito isso, as DCC continuam um problema de saúde pública em todo o mundo.

Durante os últimos quatro séculos, a especialidade da cardiologia pediátrica tem crescido imensamente, alcançando diversos marcos no cenário clínico e cirúrgico, devido, principalmente, aos incríveis avanços nas ferramentas diagnósticas de imagem cardiovascular. Isso permite o diagnóstico não invasivo em todas as idades, desde a vida fetal até a idade adulta. A modalidade relativamente nova de imagem por ressonância magnética cardíaca (RMC) fornece informações clinicamente úteis em várias situações de pacientes com DCC. As diretrizes atuais para diagnóstico e tratamento incluem ecocardiograma, RMC e cateterismo cardíaco para a maioria dos pacientes com DCC.^3^

Kozak et al.,^4^ tiraram uma “fotografia instantânea” da situação brasileira em relação ao uso da RMC em populações pediátricas, entrevistando cardiologistas da maioria dos estados, incluindo centros com diferentes níveis de atenção e volumes cirúrgicos. A “imagem” foi clara: *a RMC não está sendo plenamente utilizada*! Aproximadamente metade dos cardiologistas (52%) raramente utilizam a RMC para crianças. Ao ampliarmos esta imagem, duas camadas principais são reveladas. A primeira camada envolve o processo de mudança de práticas e da incorporação de novas tecnologias. O uso predominante da RMC ainda depende do uso para cardiomiopatias, tetralogia de Fallot pós-reparo e anomalias do arco aórtico, segundo esses dados. No entanto, a era atual da RMC em cardiologia vai além da avaliação anatômica, permitindo acesso a dados hemodinâmicos e outros dados funcionais que impactam no atendimento aos pacientes, na tomada de decisão cirúrgica e até mesmo nos fatores de risco prognóstico. A segunda camada, não surpreendentemente, é o custo da RMC, que deve ser analisado no contexto dos custos de saúde para populações com DCC. À medida que os países se desenvolvem economicamente, a carga das condições relacionadas à pobreza diminui e é substituída por necessidades de cuidados crônicos e frequentemente complexos, como as DCC. A taxa de sobrevivência e a qualidade de vida desses pacientes, desde a infância até a idade adulta, dependem da excelência do atendimento médico. Devido à sua complexidade, as lesões cardíacas cirurgicamente reparadas requerem vigilância ao longo da vida.^5^ Portanto, todos esses pontos devem ser levados em consideração no planejamento de cuidados ideais para pacientes com DCC. É caro e requer investimento ao longo da vida. No entanto, o investimento não é a solução única; há uma tríade que precisa ser abordada simultaneamente, “*pessoas, processo e tecnologia*”. Kozak et al.,^4^ demonstrou que existem três limitações principais: 1) custo (65%), 2) necessidade de sedação (60%) e 3) número insuficiente de profissionais qualificados (55%). A estas limitações interpõe-se ações para atender à tríade: o investimento em novas tecnologias na área da saúde, a necessidade de desenvolvimento profissional, e um processo para tornar a tecnologia eficaz e impactar a qualidade do atendimento. O treinamento de cardiologistas pediátricos no mundo de hoje requer uma sub-especialidade em diagnóstico por imagem, que tem avançado demasiadamente lenta, principalmente no que diz respeito ao treinamento de RMC.^6^ O aumento do número e da qualidade de profissionais aptos a executar e interpretar os resultados gerará um novo ciclo, incluindo o uso correto da RMC em pacientes com DCC, especialmente na população pediátrica.

Estes dados têm o poder de desencadear múltiplas ações no cuidado aos pacientes com DCC, a saber, alocação de recursos, capacitação profissional e mudanças nas práticas. Todas essas ações aliadas a fortes práticas de cuidado nos moverão na mesma direção, promovendo uma assistência à saúde de qualidade.
