# Supplementary material for: Imagem Cardiovascular em Cardiopatias Congênitas: Por que não Alavancar Novas Modalidades de Imagem?
Source: Arq Bras Cardiol. 2021 Feb 19;116(2):313–4. [Article in Portuguese] doi: 10.36660/abc.20201347 (PMC7909975; doi:10.36660/abc.20201347)
Supplement: Translation

# Translation: Cardiovascular Imaging in Congenital Heart Diseases: Why not Leverage New Imaging Modalities?

**Authors:** Vitor Guerra

**Affiliations:** 1 CanadaSickkids Hospital/ The Hospital for Sick Children, Department of Pediatrics, University of Toronto, Toronto, Ontario – Canada

**Keywords:** Heart Defects, Congenital/surgery, Procedures and Techniques Diagnostic, Magnetic Resonance Imaging/methods, Cardiac Catheterization

“There can be no keener revelation of a society’s soul than the way in which it treats its children.”

Nelson Mandela (8 May 1995)

Worldwide, we are living in an era burdened by congenital heart diseases (CHD).^1^ If we take a snapshot in 2018, there were 2,944,932 live births in Brazil (Tabnet, DATASUS). Considering the incidence of CHD, about 9 per 1000 live births, which has been stable across countries and populations, we would expect around 26,000 new patients with some form of CHD in our country. On the other hand, worldwide survival rate for CHD in the last 3 decades has been close to 98%.^2^ Having said that, CHD continues to be a public health problem worldwide.

In the last four centuries, the specialty of pediatric cardiology has grown tremendously, achieving several milestones in clinical and surgical scenarios, mostly due the incredible advances in cardiovascular imaging diagnostic tools. This allows non-invasive diagnosis in all ages from fetal life until adulthood. The relatively new modality of cardiac magnetic resonance imaging (CMR) provides clinically useful information in various situations in patients with CHD. Current guidelines for diagnosis and management include echocardiogram, CMR and cardiac catheterization for the majority of CHD patients.^3^

Kozak et al.^4^ took a snapshot of the Brazilian situation on the use of CMR in pediatric populations interviewing cardiologists from most states, including centers with different levels of care and surgical volumes. The big picture: *it is not being fully used*! About half of the cardiologists (52%) rarely use CMR for children. If we magnify this picture, two major layers are revealed. The first layer involves the process of changing practices and incorporating new technologies. The predominant use of CMR still relies on use for cardiomyopathies, tetralogy of Fallot post-repair, and aortic arch anomalies, according to this data. However, the current era of CMR in cardiology goes beyond anatomical assessment, allowing us to access hemodynamic and other functional data which impacts patient care, surgical decision making, and even prognostic risk factors. The second layer, not surprisingly, is the cost of CMR, which should be analysed in the context of healthcare costs for populations with CHD. As countries develop economically, the burden of poverty-related conditions diminishes and is substituted by chronic and often complex care needs, such as CHD. The survival rate and quality of life for these patients from childhood into adulthood depends on excellence of medical care. The complexity of surgically repaired heart lesions requires lifelong surveillance.^5^ Therefore, all these points should be taken in consideration when planning ideal care for patients with CHD. It is expensive, and it requires investment during a lifelong journey. However, investment is not a single solution; it is a triad which needs to be addressed simultaneously, “*people, process, and technology*”. Kozak et al.^4^ has shown that there are three major limitations: 1) cost (65%), 2) the need for sedation (60%), and 3) insufficient number of qualified professionals (55%). These address the triad: the investment in new technology in healthcare, the need for professional development, and a process to make the technology effective and impact the quality of care. The training of pediatric cardiologist in today’s world requires sub-specialty in imaging diagnosis, which has been advancing too slowly, especially with respect to CMR training.^6^ The increasing number and quality of professionals able to perform and interpret results will generate a new cycle, including correct use of CMR in patients with CHD, especially in pediatric populations.

This data has the power to trigger multiple actions in caring for patients with CHD, namely, allocation of resources, professional training, and changes in practices. All these actions coupled with strong care practices move us in the same direction, promoting high quality health care.
